# A retrospective cohort study of Libmeldy (atidarsagene autotemcel) for MLD: What we have accomplished and what opportunities lie ahead

**DOI:** 10.1002/jmd2.12378

**Published:** 2023-06-22

**Authors:** Claire Horgan, Kelly Watts, Dipak Ram, Stewart Rust, Rebekah Hutton, Simon Jones, Rob Wynn

**Affiliations:** ^1^ Royal Manchester Children's Hospital Manchester University NHS Foundation Trust Manchester UK

**Keywords:** gene therapy, metachromatic leukodystrophy, newborn screening, transplant

## Abstract

Metachromatic leukodystrophy (MLD) results from ARSA gene mutations. Affected individuals meet early milestones before neurological deterioration and early death. Atidarsagene autotemcel (arsa‐cel), an autologous haematopoietic stem cell gene therapy (HSC‐GT) product, has demonstrated sustained clinical benefits in MLD. Arsa‐cel was approved for NHS treatment in February 2022 for asymptomatic late infantile or early juvenile disease, or early symptomatic early juvenile MLD. We evaluate the impact of this approval in the largest real‐world dataset of MLD HSC‐GT. Hospital records were reviewed for all patients referred for NHS treatment following arsa‐cel approval. Information was gathered about disease phenotype, presentation, eligibility, and affected siblings. In the year following NHS approval, 17 UK MLD patients were referred for treatment. Four patients met eligibility criteria and have been treated, including 1 infant who weighed 5 kg at leukapheresis. Eleven patients failed screening: 10 symptomatic patients with late infantile disease and 1 with early juvenile disease and cognitive decline. Two further patients with later onset subtypes did not meet the approval criteria. Three out of four treated patients were diagnosed by screening after MLD was diagnosed in a symptomatic older sibling. The success of HSC‐GT for MLD has heralded a new era of hope for families affected by this devastating disease, yet currently, most patients are ineligible for treatment at diagnosis. The feasibility of apheresis in infants and the availability of a licenced, effective HSC‐GT product highlights the urgent need for newborn screening to ensure that patients can be diagnosed and treated before symptom onset.

## 1 BACKGROUND

Allogeneic haematopoietic stem cell (HSC) transplant can attenuate lysosomal storage diseases, as enzyme donated from donor‐derived leukocytes is delivered to host tissues and catabolises accumulating substrate. There is a relationship between enzyme dose delivered, and biochemical and clinical outcomes, and allogeneic HSC better prevents disease progression than reverses established disease.

Timely HSC‐GT using gene‐modified autologous HSC that delivers normal or supraphysiological enzyme levels has the potential to alter the natural history of such diseases. Early diagnosis and prompt access to gene therapy are imperative, especially in rapidly progressive diseases that involve the nervous system, since there is an obligate delay in enzyme‐competent cells engrafting this compartment. Pre‐symptomatic diagnosis is possible through newborn screening (NBS) and allows earlier treatment by harvesting HSC from young infants which can then be sent for gene therapy manufacture.

MLD is a rare, autosomal recessive, lysosomal storage disease with an estimated incidence of 1 case in 40  000 live births.^1^ The condition arises due to mutations in the ARSA gene leading to a deficiency in the enzyme arylsulfatase A (ARSA).^2^ The clinical phenotype is a consequence of accumulation of sulfatides throughout the CNS and peripheral nervous system, which results in progressive demyelination, neuroinflammation, and neurodegeneration.^3^, ^4^ Sulfatides also accumulate in visceral organs, such as the gallbladder^5^ and kidneys.^6^ Affected individuals may appear normal at birth, and meet early developmental milestones, before motor and cognitive deterioration ensues, with progressive loss of motor and cognitive functions leading to early death.^7^

The condition is classified according to age of symptom onset. Children developing symptoms before the age of 2.5 years have late infantile disease, the juvenile form is characterised by symptom onset between the age of 2.6 and 16 years (early juvenile <7 years and late juvenile from 7 to 16 years), and adult‐onset disease occurs from age 16 onwards.^7^, ^8^ The early onset forms typically have a rapidly progressive disease course, with only 50% of individuals diagnosed with the late infantile form surviving beyond 2.7 years post first symptom onset and 5‐year survival of just 25%.^9^ Recent data suggests that this is also true for older patients who have motor symptoms at presentation.^10^

Allogeneic HSC transplant has shown limited efficacy in attenuating the neurological deterioration that characterises MLD, particularly in those patients with early‐onset disease,^11^, ^12^ highlighting the unmet need in this population. Patients with juvenile and adult‐onset forms may benefit from allogeneic transplant if performed early in the disease course before the onset of symptoms,^13^, ^14^ however, peripheral neuropathy continues to be a significant issue regardless of subtype and the secondary muscle weakness that results impairs long term gross motor outcomes.^13^

Atidarsagene autotemcel (registered as Libmeldy®—hereafter referred to as ‘arsa‐cel’), an autologous HSC‐GT product based on autologous CD34^+^ stem cells transduced ex‐vivo with a lentiviral vector encoding the human ARSA gene, has demonstrated sustained clinically relevant benefits in children with early onset MLD, by preserving cognitive and motor function, and slowing demyelination and brain atrophy.^15^, ^16^ Arsa‐cel received marketing authorisation for the treatment of MLD in December 2020^17^ in the EU, in January 2021 in the United Kingdom, and was subsequently approved for NHS treatment in the United Kingdom by NICE in February 2022.^18^ These licencing criteria, however, have strict limitations in that treatment is only approved for patients who have either asymptomatic late infantile or early juvenile disease or early symptomatic early juvenile MLD, with early clinical manifestations of the disease, with maintained ability to walk independently and before the onset of cognitive decline, see Table 1. Patients falling outside of these indications are not eligible and these children and their families must endure the natural history of the disease and its devastating consequences.

**TABLE 1:** Licenced indication for treatment with Libmeldy (highlighted in green).

MLD subtypes	Late infantile	Juvenile		Adult
		Early juvenile	Late juvenile	
Age of symptom onset	< 30 months	30 months to <7 years	7–16 years	17 + years
Pre‐symptomatic	Without clinical manifestations	Without clinical manifestations	x	x
Early symptomatic	x	GMFC‐MLD score ≤1 and FSIQ ≥ 85	x	x

Abbreviations: FSIQ, full‐scale intelligence quotient. GMFC‐MLD, gross motor function classification‐metachromatic leukodystrophy.

We evaluate the impact NHS arsa‐cel approval has had on UK patients with early onset MLD in the largest real‐world dataset of a gene‐therapy‐treated MLD cohort outside of clinical trials. Multi‐centre natural history studies have provided a convincing picture of the different types of MLD^8^, ^10^ and our treatment study assesses how gene therapy, administered under the current licencing indications, may impact this. Arsa‐cel must be administered in a Qualified Treatment Centre (QTC) experienced in HSC transplantation and the management of leukodystrophies. The licence is independent of treatment centre selection but the choice of a limited number of treatment centres ensures consistency and quality of treatment. Royal Manchester Children's Hospital (RMCH) is the only arsa‐cel QTC in the United Kingdom, hence our dataset is likely to be a complete reflection of the current HSC‐GT landscape of MLD within the UK based on referrals.

## 2 METHODS

Hospital records were reviewed for all UK patients referred (*n* = 17) to RMCH for consideration of HSC‐GT since Libmeldy was approved for NHS treatment in February 2022. Information was gathered about disease phenotype and clinical presentation along with eligibility for HSC‐GT and whether any siblings were affected. Patients were screened against the licencing criteria, and where appropriate (in the absence of clear features at referral that made them ineligible), were reviewed by a multi‐disciplinary expert panel including metabolic, neurology, and neuropsychology representatives to confirm their eligibility status.

**FIGURE 1:**
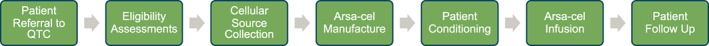
The arsa‐cel patient treatment journey. Patient referred to Qualified Treatment Centre (QTC) where eligibility is assessed. If eligibility confirmed patient undergoes leukapheresis and stem cell collection. The product is sent to the manufacturing site for arsa‐cel production. Once the quality assured product is returned to QTC, the patient undergoes conditioning therapy prior to the product being infused.

Patients meeting eligibility criteria were referred immediately to the stem cell transplant team who is responsible for cell mobilisation protocols, and for the clinical care of the patient during mobilisation, leukapheresis, conditioning, and reinfusion of the genetically modified HSC, see Figure 1 for patient treatment journey. Patients not meeting eligibility criteria were discharged back to their referral centre for palliative care. Patients were consented to provide data for outcomes analysis and information was gathered directly.

## 3 RESULTS

In the 1 year period, from February 2022 to February 2023, since Libmeldy received approval for NHS reimbursement, 17 UK patients with MLD have been discussed with the team at RMCH for consideration of HSC‐GT treatment. Four patients (21%), including 1 patient who was screened at birth due to a previously affected sibling and weighed approximately 5 kg at the time of leukapheresis, met eligibility criteria (see Table 2). Three of the four treated patients were identified by screening after MLD was diagnosed in a symptomatic older sibling.

**TABLE 2:** Demographics and eligibility data of UK patients meeting criteria for MLD HSC‐GT.

Phenotype	Symptoms	Age at referral	Weight (kg) at apheresis	Affected sibling	Duration from referral received to HSC‐GT infusion (days)
Late infantile	None	1 year	7.76	Yes	97
Late infantile	None	Following testing at birth	5.52	Yes	113^a^
Early juvenile	Mild cognitive impairment FSIQ 85, able to walk independently	6 years	23.78	Yes	76
Early juvenile	Motor deterioration with altered balance and ataxic gait, able to walk independently FSIQ 101	6 years	23.6	No	89

Abbreviation: FSIQ, full‐scale intelligence quotient.

a Original infusion postponed 2 weeks due to septic episode and patient requiring 48‐h admission to paediatric intensive care unit.

All four have undergone successful mobilisation, leukapheresis, manufacture, and Libmeldy infusion.

Thirteen out of the 17 patients were ineligible on screening, including 10 patients with late infantile disease who were symptomatic on eligibility assessment, one with early juvenile disease and cognitive decline (full‐scale intelligence quotient (FSIQ) 70), 1 with late juvenile disease and cognitive decline (FSIQ 51), and a further symptomatic patient with adult‐onset disease. Table 3 gives more detail about the phenotype and symptoms of these patients.

**TABLE 3:** Screened UK patients who were referred to QTC but failed to reach eligibility criteria for MLD HCT‐GT.

Age at referral	Phenotype	Symptoms
2.5 years	Late infantile	Not walking‐ was walking and stopped, signs of spasticity, regression of skills from age 2
6 years	Early juvenile	Walking independently but familial and social concerns re memory retention, FSIQ 70 (<85)
3 years	Late infantile	Not walking‐ had previously walked but regressed
3 years	Late infantile	Walking but with very ataxic gait and increased tone, frequent falling, within 3 weeks of consultation stopped walking
3 years	Late infantile	Just able to walk but with unsteady gait and spasticity
17 months	Late infantile	Delayed motor development, increased tone in lower limbs
26 months	Late infantile	Neurological regression with loss of skills, not walking
9 years	Late juvenile	Cognitive regression with FSIQ51, memory retention issues, loss of speech
35 months	Late infantile	Neurological regression with loss of skills, not walking
23 years	Adult onset	Behavioural change, loss of skills
6 years	Late infantile	Previous child deceased, child already symptomatic, speech and language delay and cognitive impairment, delayed presentation as parents knew no treatment available for first child so had assumed the same
23 months	Late infantile	Loss of skills and increased lower limb tone
23 months	Late infantile	Still not walking due to increased tone and spasticity in lower limbs

In summary, we have treated 4 eligible early‐onset MLD patients out of the 17 NHS patient referrals received. In addition, we have demonstrated the feasibility of mobilisation, leukapheresis, product manufacture, and infusion in patients with weights as low as 5.5 kg. Despite this, the vast majority (79%) of children referred for HSC‐GT were deemed ineligible due to advanced disease.

## 4 DISCUSSION

Despite the hope afforded by HSC‐GT for patients with MLD, the substantial number of ineligible patients referred to RMCH, is a powerful reminder of the of urgent need to implement a national NBS program for the condition. A recent qualitative MLD caregiver survey conducted in the UK strongly supports the need for such NBS programs.^19^ This survey includes families with an already affected child who have benefited from prenatal or screening at birth of further children and subsequent HSC‐GT if also affected.

We acknowledge that the number of patients referred for consideration of Arsa‐cel treatment on the NHS in this first year exceeds the known incidence^1^ and hypothesise that this may be explained by a lack of familiarity amongst referring physicians with the strict licencing criteria. More education for referring physicians is needed to manage expectation and avoid giving false hope to families whilst these criteria remain in place in the absence of NBS.

Studies have demonstrated the presence of elevated sulfatides associated with MLD can be detected from dried blood spots.^19^, ^20^ Proof of concept of this NBS approach has been reported in 80  000 newborns in a pilot study in Germany.^21^ A recent US study has also confirmed the feasibility of NBS for MLD^22^ using a two‐tiered approach, with elevated sulfatides triggering a second test to measure ARSA activity. This approach resulted in nearly 100% assay specificity when tested on a cohort of 27  335 newborns.

A condition is suitable for a screening program if it strongly fulfils the Wilson and Junger principles.^23^ Our experience, together with the availability of stem cell gene therapy and NBS for the disorder, proves that MLD is an ideal candidate (see Table 4). Furthermore, a recent UK study evaluating the cost‐effectiveness of a potential MLD NBS screening program for the NHS has shown a potential 790 quality‐adjusted life years (QALYs) gained compared with no screening, and a corresponding incremental cost‐effectiveness ratio below £30 000 for each QALY gained.^24^ This suggests that NBS for MLD will therefore be an effective use of NHS resources driven by the substantive QALY gain for patients treated pre‐symptomatically.

**TABLE 4:** Wilson and Junger screening principles applied to MLD.

Wilson and Junger principle	Met by an MLD newborn screening program?
The condition should be an important health problem	YES Estimated birth prevalence 1.4–1.8 in 100 000 (29) Devastating impact on patients and families, may affect multiple individuals within a family
2 There should be an accepted treatment for patients with recognised disease	YES Atidarsagene autotemcel (Arsa‐cel) stem cell gene therapy approved by EMA (Europe) and NICE (United Kingdom) for early onset phenotypes
3 Facilities for diagnosis and treatment should be available	YES Proof of concept of NBS for MLD on dried blood spot samples of 80 000 newborns in Germany (21) Success of recent large US pilot study demonstrates feasibility of NBS for MLD with exceptionally low false positive rate (0.0037%) with two‐tier screening (22) HSC‐GT available at qualified treatment centres
4 There should be a recognisable latent or early symptomatic stage	YES Patients appear normal at birth and meet early developmental milestones before symptom onset
5 There should be suitable test or examination	YES First tier: sulfatide assay Second tier: ARSA enzyme assay and genetic confirmation
6 The test should be acceptable to the population	YES Test can be incorporated into existing dried blood spot heel prick testing NBS program
7 The natural history of the condition, including development from latent to declared disease should be adequately understood	YES Progressive motor and cognitive decline, more rapid in earlier forms and patients with motor symptoms at presentation
8 There should be an agreed policy on whom to treat as patients	YES Early and prior to symptom onset is best Late infantile patients must be asymptomatic and can therefore ONLY be detected by NBS in the absence of a family history Early juvenile patients must maintain cognitive function FSIQ > 85 and the ability to walk independently
9 The cost of case‐finding (including diagnosis and treatment of patients diagnosed) should be economically balanced in relation to possible expenditures on medical care as a whole	YES Stem cell gene therapy process is expensive, but a single one‐off cost compared to the years of intensive palliative care and family support for patients with such complex needs Incremental QALY gain of around 790 years versus no screening in UK study showing the cost effectiveness of MLD NBS to the NHS (24)
10 Case finding should be a continuous process and not a ‘once and for all’ project	YES Formal incorporation into established NBS programs would ensure that this is the case

A formal application to add MLD to the UK newborn blood spot program was made in December 2021 to the national screening committee marking an important milestone for the MLD community.^25^ During the lengthy approval process, likely to take several years, patients will continue to be diagnosed with disease too advanced to benefit from HSC‐GT. This will result in families enduring the consequences of MLD, including deterioration and early death of their child and the devastating psychological and financial burden for the whole family.^26^ Novel NBS evaluation algorithms have been proposed to try and prioritise inherited metabolic disorders, such as MLD, for inclusion on expanded NBS programs across Europe, highlighting the need to make timely decisions and reduce the gap between treatment approval and national NBS.^27^, ^28^ The severity and rapid progression of MLD together with the availability of a treatment strategy that can change prognosis if patients are treated pre‐symptomatically makes this issue even more pertinent, and a collaborative approach to mandating cost‐effective NBS is required.

The rarity and complexity of MLD, whose typical presentation can overlap with other neuromuscular disorders, causes diagnostic delays frequently described by families affected by the disorder.^26^ While education of healthcare professionals and the wider public may help with the importance of prompt recognition, and urgent referral of early onset MLD patients to a specialist center or QTC, the reality is that in the absence of prior family history, patients with the late infantile variant are unlikely to be diagnosed early enough and be eligible for HSC‐GT.

NBS will prevent delays in diagnosis and the associated burden faced by families, allowing families time to process this diagnosis and prepare for HSC‐GT.

In conclusion, our largest real‐world dataset of patients treated with arsa‐cel autologous HSC‐GT for early onset MLD highlights the importance of a prompt diagnosis and referral to maximise the potential treatment benefit. NBS has the potential to reduce the current high proportion of ineligible patients by ensuring rapid diagnosis at birth and timely HSC‐GT before symptom onset while being aware of the stark reality patients who are ineligible for treatment will succumb to their disease due to a late diagnosis. We must lobby together to fast‐track an MLD UK NBS program to transform the therapeutic landscape of the condition, allowing this new era of stem cell gene therapy to achieve its full potential.

## AUTHOR CONTRIBUTIONS

Rob Wynn, Simon Jones, and Claire Horgan conceived and designed the study. Claire Horgan, Kelly Watts, Dipak Ram, Stewart Rust, Rebekah Hutton, Simon Jones, and Rob Wynn collected and supplied all institutional data. CH wrote the manuscript. All authors reviewed, edited, and approved the submitted manuscript.

## FUNDING INFORMATION

The authors confirm independence from the sponsors; the content of the article has not been influenced by the sponsors.

## CONFLICT OF INTEREST STATEMENT

Claire Horgan declares that she has no conflicts of interest. Kelly Watts declares that she has no conflicts of interest. Dipak Ram declares that he has no conflicts of interest. Stewart Rust declares that he has no conflicts of interest. Rebekah Hutton declares that she has no conflicts of interest. Simon Jones has received grant funding and licencing revenue from Orchard Therapeutics. Rob Wynn has received grant funding and licencing revenue from Orchard Therapeutics.

## INFORMED CONSENT

All procedures followed were in accordance with the ethical standards of the responsible committee on human experimentation (institutional and national) and with the Helsinki Declaration of 1975, as revised in 2000. Informed consent was obtained from all patients. All patients are consented to data collection and sharing. This is a service outcome paper and not a research study so specific ethics approval not required.

## Data Availability

All data supporting the results are available within the manuscript content.
